# Perioperative immune responses in cancer patients undergoing digestive surgeries

**DOI:** 10.1186/1477-7819-7-7

**Published:** 2009-01-12

**Authors:** Masashi Ishikawa, Masanori Nishioka, Norikazu Hanaki, Takayuki Miyauchi, Yutaka Kashiwagi, Hiromi Ioki, Akihiro Kagawa, Yoichi Nakamura

**Affiliations:** 1 Department of Surgery, Tokushima Red Cross hospital103 Irinokuchi, Komatsushima city, Tokushima, Japan; 2 Department of Surgery, National Kochi Hospital, Japan

## Abstract

**Background:**

Th1/Th2 cell balance is thought to be shifted toward a Th2-type immune response not only by malignancy but also by surgical stress. The aim of this study was to estimate perioperative immune responses with respect to the Th1/Th2 balance in patients with gastrointestinal cancer.

**Methods:**

Ninety-four patients who underwent abdominal surgeries were divided into three groups: gastric resection (n = 40), colorectal resection (n = 34) and hepatic resection (n = 20). Twelve patients undergoing laparoscopic cholecystectomy and 20 healthy subjects were served as control groups. Intracellular cytokine staining in CD4+ T lymphocytes was identified to characterize Th1/Th2 balance. Th1/Th2 balance was evaluated before operation and until postoperative days (POD) 14.

**Results:**

The preoperative Th1/Th2 ratio was significantly lower in patients with malignancy compared with control. The Th1/Th2 ratio of patients in all groups decreased significantly postoperatively. Th1/Th2 balance on POD 2 in patients with malignancy was significantly decreased compared to patients with laparoscopic cholecystectomy, but there were no significant differences among the four groups on POD 14.

**Conclusion:**

Patients with malignancy showed an abnormal perioperative Th1/Th2 balance suggesting predominance of a type-2 immune response. Major abdominal surgeries induce a marked shift in Th1/Th2 balance toward Th2 in the early postoperative stage.

## Background

Immunity to malignancy is influenced not only by CD8+ T cells, but also by the function of CD4+ T helper (Th) lymphocytes, which are important in cell-mediated and humoral immunity [1]. Since the initial description by Mosmann et al. [2] of subclasses of CD4+ helper T cells (Th) that differ in cytokine secretion, immune responses have been classified into type 1 responses that provide cell-mediated immunity, and type 2 responses that support B cell functions and the humoral immune response. Exposure of naïve Th cells to certain antigens and cytokines causes CD4+ T cells to assume one of these two distinct phenotypes. Th1 cells produce predominantly interferon-γ (IFN-γ) and interleukin-2 (IL-2), whereas Th2 cells secrete predominantly IL-4, IL-6 and IL-10 [3].

Several studies have shown reduced secretion of Th1 in bulk cultures of peripheral blood mononuclear cells (PBMC) from advanced cancer patients [4,5]. However, a few studies of Th1/Th2 balance have been performed on large numbers of patients with abdominal cancer [6,7]. The first objective of the present study was to estimate roles of these two CD4+ subsets in anti-tumor immunity.

It has been reported that the Th1/Th2 cell balance is shifted toward a Th2-type immune response not only by malignancy but also by surgical stress [8-10]. Decker et al. [11] have shown that surgical stress induces a shift in the Th1/Th2 cell balance, suggesting a down-regulation of cell-mediated and up-regulation of antibody-mediated immunity commensurate with surgical trauma. However, the extent to which surgical stress influences the Th1/Th2 balance is unknown. The second objective of this study was to assess changes in the Th1/Th2 balance in patients undergoing surgical stress, and to clarify whether a shift in the Th1/Th2 balance can be used to facilitate comparisons of different abdominal surgeries.

## Patients and methods

Ninety-four consecutive patients (55 males, 39 females; mean age of 66 ± 11 years) underwent abdominal surgery for the first time at our clinic between April 2000 and April 2002. The experimental protocol was approved by the Research Committee of National Kochi Hospital. All patients were informed of the nature and risk of this study, and written informed consent was obtained. The criteria for inclusion were major surgical tumor resection (digestive tract or liver cancers) and expected duration of operation of 3 hours or more. The patients were divided into the following three groups according to surgical procedure. Forty gastric resections for gastric cancer (24 males, 16 females; mean age of 65 ± 13 years), 34 colorectal resections for colorectal cancer (19 males, 15 females; mean age of 66 ± 15 years) and 20 hepatic resection for liver cancer (8 hepatocellular carcinomas, 5 liver metastases and 7 biliary tract carcinomas) (12 males, 8 females; mean age of 66 ± 8 years) were performed, respectively (Table 1).

**Table 1:** Details of the procedures

Disease	Case	Male:female	Surgical procedure	Operating time (min)	Intraoperative bleeding (ml)
Gastric cancer	40	24:16	Gastric resection (40)	223 ± 55	267 ± 212
Colon cancer	34	19:15	Intestinal resection (34)	220 ± 71	325 ± 336
Hepatobiliary cancer	20	12:8	Partial resection (8)	365 ± 85	761 ± 759
HCC	8	12:8	Subsegmental resection (2)	365 ± 85	761 ± 759
Liver metastasis	5	12:8	Segmental resection (3)	365 ± 85	761 ± 759
Ca of biliary tract	7	12:8	Lobectomy (7)	365 ± 85	761 ± 759
Cholelithiasis	12	6:6	Laparoscopic cholecystectomy (12)	116 ± 45	46 ± 180

Twelve laparoscopic cholecystectomy (LC) (6 males, 6 females; mean age of 58 ± 14 years) and 20 healthy subjects (10 males and 10 females; mean age of 60 ± 12 years) served as the control groups. The patients with malignancy were divided into four groups corresponding to the four stages of disease (stage I (n = 28), stage II (n = 36), stage III (n = 22), stage IV (n = 8), respectively) by UICC classification. The duration of operation and operative blood loss for each procedure were 223 ± 55 min and 267 ± 212 ml for gastric resection, 220 ± 71 min and 325 ± 336 ml for colorectal resection, 365 ± 85 min and 761 ± 759 ml for hepatic resection and 116 ± 45 min and 46 ± 80 ml for LC, respectively. Ten patients (5 [25%] hepatic resections, 2 [5%] gastric resections, 3 [9%] colorectal resections) developed postoperative complications (4 abdominal abscesses, 4 leakages and 2 pneumonia). These major infectious complications typically occurred on POD 4 or 5 (range 3–7 days). All patients had a relatively uneventful recovery within POD 30 (range 15–25 days). No patients with laparoscopic cholecystectomy developed postoperative complications.

### Flow cytometric analysis of intracellular IFN-γ and IL-4

Blood sampling was performed before surgery, and on POD 2 and 14. CBC and leukocyte differential analyses were performed with an automated cell counter (Sysmex SE-9000, Kobe, Japan). The proportion of CD4+ lymphocytes producing IFN-γ, IL-2, IL-4 and IL-6 were measured by flow cytometry (Beckman Coulter, Inc., Florida, Miami, USA) as described by Openshaw et al, [12]. Briefly, 1 ml of each blood sample was treated immediately with 10 ng/ml of Brefeldin A (Sigma B7651), kept at ambient temperature, and prepared within 2 hours. Peripheral blood lymphocytes were harvested, washed, and resuspended at 10^5^–10^6^/ml and stimulated with PMA 50 ng/ml (Sigma P8139) plus ionomycin (Sigma 10634) 500 ng/ml. After a wash and 10-min incubation in PBS/BSA/saponin, cells were incubated with anti-CD4 monoclonal Ab and anti-IFN-γ (DAKO, RG285, Denmark), anti-IL-2 (DAKO, RG202), anti-IL-4 (DAKO, RG204) or anti-IL-6 (DAKO, RG206) for 30 min before adding an equal volume of 4% formaldehyde fixative. After washing and incubating with PBS/BSA/saponin for 10 min, cells were incubated with for 30 min, respectively. Results were analyzed using the XL/XL-MCL system and were calculated as a ratio of the percent IFN-γ-producing (Th1) cells to IL-4-producing (Th2) cells.

### Statistical analysis

Values for results are presented as means ± SD. Student's *t *test was used for comparison of continuous variables. Differences between groups with respect to time were compared using the analysis of variance (ANOVA) for repeated measures. A p value of < 0.05 was considered significant.

## Results

**Figure 1:**
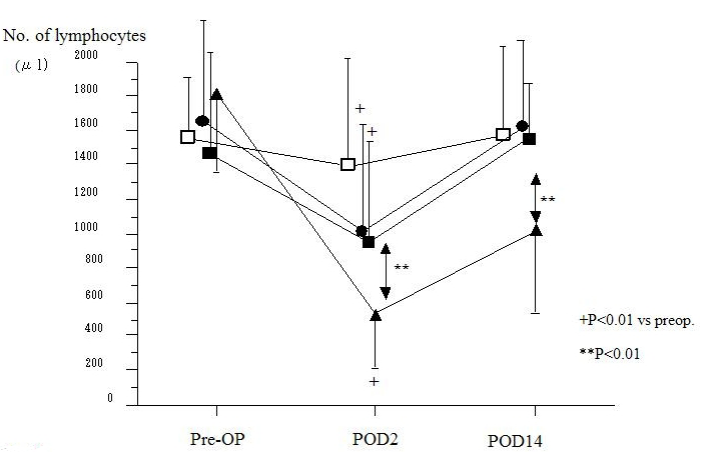
**Lymphocyte counts for patients**. (Black squares – gastric resection (n = 40), black circles – colorectal resection (n = 34), black triangles – hepatic resection (n = 20), white squares – laparoscopic cholecystectomy(n = 12)). Lymphocyte count in all groups decreased significantly after surgery. The lymphocyte counts for patients with hepatic resection became significantly lower than those in the gastric and colorectal resection groups after surgery.

The lymphocyte count in all patients decreased significantly after surgery, reaching a nadir of almost one-third of baseline on POD 2 (Fig. 1). There was no significant difference in the lymphocyte count among the four groups including the LC group before surgery. Patients with hepatic resection presented with 531 ± 24 cells/μl on POD 2, then rebounded by POD 14 to 1017 ± 494 cells/μl. However, the lymphocyte count in patients with hepatic resection was significantly lower than in those with gastric and colorectal resection on POD 2 and 14.

**Figure 2:**
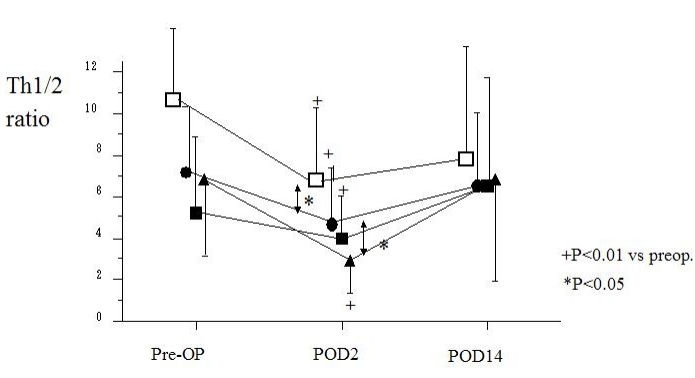
**Changes in the Th1/2 ratio in surgical patients with respect to operative procedure**. (Black squares – gastric resection (n = 40), black circles – colorectal resection (n = 34), black triangles – hepatic resection (n = 20), white squares – laparoscopic cholecystectomy(n = 12)). The Th1/2 ratio in all groups decreased significantly on POD 2 and significant differences were noted between malignancy group and LC group. However, the ratio in all groups recovered to preoperative levels on POD 14.

The preoperative percentage of IL-4-producing T cells from PBMC of patients with malignancy was significantly higher than in the control groups, while patients who underwent LC showed no significant differences from the healthy controls. In contrast, the preoperative percentages of IL-2, IFN-γ and IL-6-producing T cells in all patient groups were similar to the healthy control group. Therefore, the preoperative ratio of the percentage of IFN-g-producing T cells (Th1) to IL-4-producing T cells (Th2) was significantly higher in healthy controls and patients who underwent LC than in those who underwent operations for malignancy, the means being 11.3 ± 4.3, 10.8 ± 5.6 and 6.8 ± 4.2 (p < 0.01), respectively. There were no significant differences in the preoperative ratio of Th1 to Th2 among gastric, colorectal and hepatic resection groups, the mean being 5.2 ± 3.6 in patients with gastric cancer, 7.2 ± 3.1 in those with colorectal cancer and 6.6 ± 4.0 in those with hepatic cancer (Fig. 2). Preoperatively, malignancy groups showed a significantly higher percentage of T cells producing IL-4 than that of LC and healthy control groups. Interestingly, no significant differences were observed according to staging in patients with malignant disease: 6.9 ± 3.5 in stage I, 6.5 ± 3.7 in stage II, 6.6 ± 3.1 in stage III, 5.5 ± 2.6 in stage IV.

**Figure 3:**
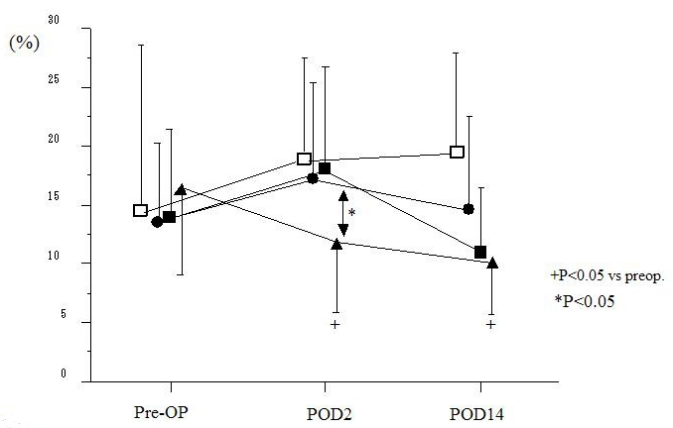
**Perioperative changes of measurements of IFN-γ in surgical patients**. (Black squares – gastric resection (n = 40), black circles – colorectal resection (n = 34), black triangles – hepatic resection (n = 20), white squares – laparoscopic cholecystectomy(n = 12)). There were no significant differences in the percentage of CD4+ IFN-γ+T cells among all groups prior to surgery. Significant decrease, however, in the postoperative percentage of CD4+ IFN-γ+T cells were not seen other than the reduction on POD 2 in the hepatic resection group.

**Figure 4:**
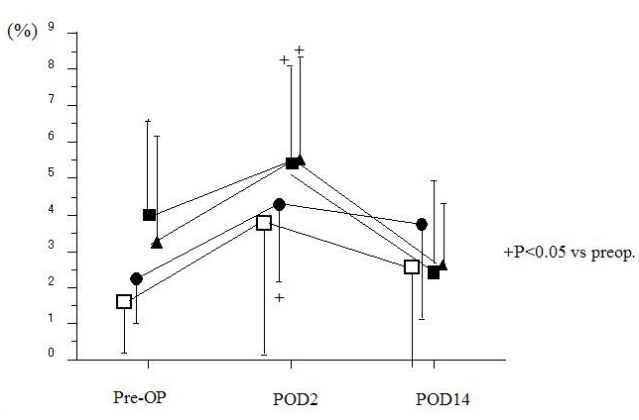
**Perioperative changes of measurements of IL-4 in surgical patients**. (Black squares – gastric resection (n = 40), black circles – colorectal resection (n = 34), black triangles – hepatic resection (n = 20), white squares – laparoscopic cholecystectomy(n = 12)). There were significant differences in the percentage of CD4+ IL-4+T cells between malignancy group and LC groups prior to surgery. The postoperative percentage of CD4+ IL-4+T cells in all groups significantly increased on POD 2 compared with before operation.

Postoperatively, the ratio of Th1 to Th2 decreased in all groups (4.5 ± 3.0 in malignancy groups, 6.7 ± 4.1 in LC groups on POD 2) (Fig. 2). The ratio of Th1/2 in patients with malignancy markedly decreased to 4.3 ± 2.1 in gastric resection, 4.9 ± 2.6 in colorectal resection and 2.9 ± 1.6 in hepatic resection on POD 2, with significant differences (p < 0.05) compared to patients undergoing LC. However, these ratios recovered to preoperative levels on POD 14 in all groups. There were no significant differences in the percentage of CD4+IFN-γ+ T cells among all groups prior to surgery (14.0 ± 8.5% in gastric resection, 13.6 ± 6.6% in colorectal resection, 16.3 ± 8.3% in hepatic resection and 14.6 ± 15.6% in LC). Significant changes in the postoperative percentage of CD4+IFNγ+ T cells were not seen other than a reduction on POD 2 in the hepatic resection group (11.7 ± 6.9%, P < 0.05) (Fig. 3). In contrast to CD4+IFNγ+ T cells frequencies, the percentage of CD4+IL-4+ T cells in all groups significantly increased on POD 2 (5.1 ± 2.7% in gastric resection, 4.5 ± 2.7% in colorectal resection, 5.5 ± 2.8% in hepatic resection and 3.2 ± 3.0% in LC) compared with before surgery (4.1 ± 2.5% in gastric resection, 2.6 ± 1.5% in colorectal resection, 3.3 ± 2.6% in hepatic resection and 1.6 ± 1.3% in LC) (Fig. 4).

There were no significant differences in the percentage of CD4+IL-2+ T cells among all groups prior to surgery (17.2 ± 16.6% in gastric resection, 12.7 ± 11.0% in colorectal resection, 16.2 ± 12.7% in hepatic resection and 8.0 ± 5.8% in LC). No significant differences in the percentage of CD4+IL-2+ T cells among all groups after surgery were found. On the other hand, there were also no significant differences in the percentage of CD4+IL-6+ T cells among all groups prior to surgery (39.2 ± 28.9% in gastric resection, 32.1 ± 23.3% in colorectal resection, 40.0 ± 24.9% in hepatic resection and 30.3 ± 37.0% in LC), showing no significant differences among all groups after surgery.

**Figure 5:**
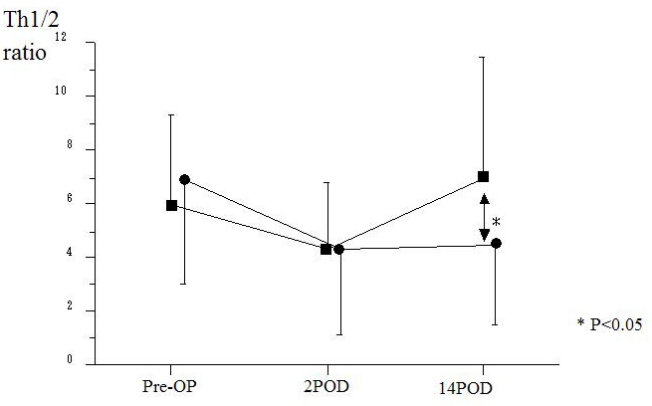
**Changes in Th1/Th2 ratio in surgical patients with malignancy from the viewpoint of postoperative complications**. (Complication (-) n = 84, complication (+) n = 10) The patients with postoperative complications showed significantly lower Th1/2 ratios on POD 14 although there were no significant differences in the two groups before operation or on POD 2.

In the malignancy group there were no significant differences between patients with and without postoperative complications prior to surgery and on POD 2 (4.6 ± 2.5 *vs*. 4.4 ± 3.2), but the Th1/2 ratio in 10 patients with postoperative complications was 3.5 ± 2.0 on POD 14, significantly lower than in patients without postoperative complications (7.2 ± 4.4, p < 0.05) (Fig. 5). The percentages of CD4+IFN-γ+T cells in patients with postoperative complications decreased significantly to 11.4 ± 6.1% on POD 14 compared to patients without postoperative complications (14.6 ± 6.6%). In contrast, the percentage of CD4+ IL-4+ T cells in patients with and without complications on POD 2 exhibited the opposite trend (5.3 ± 1.5 *vs*. 4.3 ± 1.0%, for patients with and without complications, respectively, p < 0.05).

## Discussion

Some previous studies have shown that cell-mediated but not humoral immunity is impaired in cancer patients [13]. However, it has not been determined whether this impairment is more generally associated with the presence tumors. Surprisingly, among patients with cancer in the present study, there were no differences in the Th1/Th2 ratio between the patients with early cancer and advanced cancer. It therefore seems that, in advanced cancer patients, the shift towards Th2 might be due to altered representation of subsets as well as a change in capacity of cells to secrete cytokine. However, Pellegrini et al. [4] reported that serum levels of IL-4 in patients with stage I disease were significantly higher than control subjects and positively correlated with disease stage. Therefore, they also examined cytokine produced by tumor-draining lymphocytes from lymph nodes and reported that when tumor cells infiltrate the lymph node, the generation of Th cells in the lymph node environment is shifted towards a Th2-type immune response. The ability of IL-4 to inhibit IL-2 gene transcription has been confirmed by other researchers [14,15], suggesting that the elevation of IL-4 production during an ongoing immune response to tumor can down-regulate Th1 cytokine production. Therefore, in gastrointestinal cancer patients, the initial tumor establishment may arise from, or be accompanied by, a reduced Th1/Th2.

Hensler et al. [16]reported that during the early postoperative course, major surgery resulted in a severe defect of T cells to proliferate and to secrete cytokines characteristic of both Th1 and Th2 phenotypes. They also reported that during the late postoperative course (day 5), production of IL-2 and IFN-γ had increased, reaching levels similar to those observed before surgery. O'Sullivan et al., [14] demonstrated that peripheral blood mononuclear cells from trauma patients examined 1 to 14 days after injury produced significantly less IFN-γ than those from healthy control subjects, but production of IL-4 was increased. With respect to lymphocyte numbers, we found that lymphocyte numbers were depressed on POD 2 after major surgery, and recovered within the first postoperative week. Both the reduction in lymphocyte numbers and their subsequent recovery varied with the extent of surgical procedure. Some studies have found decreases in both CD4+ and CD8+ lymphocytes following major surgery [17], while other studies on gastric cancer patients revealed a significant decrease in numbers of CD8+ but not CD4+ lymphocytes [18] although our study only investigated bulk lymphocyte numbers.

The present findings showed that the Th1/Th2 balance in the LC group as well as malignancy group decreased on POD 2, but the decrease in the LC group was slight on POD 2. The increase percentage of CD4+IL-4+ T cells in all groups resulted in the observed decrease in the Th1/Th2 ratios. Interestingly, there were no significant differences on POD 14 among all groups corresponding with the finding of O'Sullivan et al [14].

Two important questions remain to be answered; whether a shift in the Th1/Th2 balance can be used to facilitate comparisons of different surgical procedures, and the clinical significance of the surgery-induced shift in the Th1/Th2 balance. The present findings confirm the findings of Decker et al., [10] who demonstrated that the Th1/Th2 balance illustrated by the IL-4/IFN-γ and CD23/HLA-DR ratios was markedly different between LC and open cholecystectomy. It was shown that down-regulation of the Th1 immune response makes patients more susceptible to infections with viruses and intracellular bacteria. However, no study has examined to what extent operative procedures in major abdominal surgeries influence the Th1/Th2 balance. To our knowledge, the present study is one of the first to provide information on the Th1/Th2 ratio during the early and delayed period after different major abdominal surgeries from a rather large patient sample. The Th1/Th2 balance in patients undergoing hepatic resection decreased significantly as compared with those in gastric resection and colorectal resection on POD 2 but a significant difference was not noted between the three groups preoperatively or later in the postoperative course (POD 14).

The present study also showed that a shift toward a Th2 immune response continued until POD 14 in patients who developed postoperative complications. There was no significant difference in the preoperative and POD 2 Th1/Th2 balance, regardless of the presence of postoperative complications. The first clinical signs of major postoperative complications were invariably preceded by a significant decline in Th1/Th2 balance after major abdominal surgeries. Therefore, these data support a causative relationship between the post-operative severity of immunosuppressive sequelae and the patient's susceptibility to infectious complications. However, it is difficult to predict the occurrence of postoperative complications from the ratio of the Th1/Th2 balance in patients prior to operation [19,20]. Post-surgical immunosuppression may be favored by the preoperative clinical-biological status of patients and the extent of surgical trauma such as hormonal changes evoked by stress, hemorrhage, transfusion and duration of the operation. In the present study, the patient's Th1/Th2 ratio before surgery or on POD 2 was not an associated factor in predicting postoperative complications, but the percentage of CD4+IL-4+ T cells in patients with postoperative complications was significantly higher than in patients without complications. Therefore, the increased percentage of Th2 cells may predict the occurrence of postoperative complications. On the other hand, van Sandick et al. [17], demonstrated that preoperative IFN-γ production acted as an independent predictive variable for the occurrence of postoperative major infection. Davis et al. [20], recently reported on the possible role of the IFN-γ receptor 1 gene in predicting major infection after operations. As severe trauma such as hepatic resection and occurrence of postoperative complications were associated with decreased numbers of IFN-γ-producing T cells, a deficiency in the type 1 response may play a key role in immune suppression of such patients, as reported in this study [9,21].

## Conclusion

The present study has demonstrated that the elevation of Th2 subset in patients with stage I cancer as compared with healthy subjects or patients with cholecystolithiasis suggests that the tumor-bearing state may induce a switch from a Th1-type to a Th2-type cytokine profile, although further research is necessary. Although there is an increase in the representation of Th2 cells among CD4+ peripheral lymphocytes in patients with gastrointestinal cancer, there is no corresponding decrease in the frequency of Th1 cells.

Impaired host immunity or excessive stress after major surgery often increases susceptibility to infection. These results suggest that the extent of postoperative immune depression is related to the extent of surgical tissue resection. The determination of Th1/Th2 balance may be of help in evaluating different surgical procedures and properly monitoring patients post-surgery.

## Competing interests

The authors declare that they have no competing interests.

## Authors' contributions

MI was involved in the design of the study and writing of the manuscript. MN and NH assembled the data. TM and YK performed the statistical analysis. HI and HK performed the study of flowcytometry. YN designed the study. All authors read and approved the final manuscript.
